# Reconstruction of cervico-thoracic defect with bipedicled deep inferior epigastric perforator free flap following resection of a giant recurrent thyroid tumor: a case report and review of literature

**DOI:** 10.1093/jscr/rjad491

**Published:** 2023-09-05

**Authors:** Abraham Zavala, María I Vargas, Walter Ayala, Antonio Muñoz, Miguel A Chávez, Jesús López, Ricardo Delgado

**Affiliations:** Department of Plastic and Reconstructive Surgery, Instituto Nacional de Enfermedades Neoplásicas, Lima 15038, Peru; Department of Plastic and Reconstructive Surgery, Instituto Nacional de Enfermedades Neoplásicas, Lima 15038, Peru; Department of Plastic and Reconstructive Surgery, Instituto Nacional de Enfermedades Neoplásicas, Lima 15038, Peru; Department of Breast and Soft Tissue Surgery, Instituto Nacional de Enfermedades Neoplásicas, Lima 15038, Peru; Department of Plastic and Reconstructive Surgery, Instituto Nacional de Enfermedades Neoplásicas, Lima 15038, Peru; Department of Plastic and Reconstructive Surgery, Instituto Nacional de Enfermedades Neoplásicas, Lima 15038, Peru; Department of Plastic and Reconstructive Surgery, Instituto Nacional de Enfermedades Neoplásicas, Lima 15038, Peru

**Keywords:** bipedicled DIEP flap, reconstructive surgery, cervico-thoracic reconstruction, microsurgery

## Abstract

The bipedicled Deep Inferior Epigastric Perforator (DIEP) flap, originally described and primarily utilized in autologous breast reconstruction for specific cases, has expanded its applications to encompass diverse anatomical regions in recent years. This report presents the case of a 69-year-old woman with a recurrent giant thyroid tumor who underwent surgical resection, resulting in a large cervico-thoracic defect effectively reconstructed using a bipedicled DIEP flap. The patient’s postoperative recovery was uneventful, and the follow-up assessments revealed a healthy, well-perfused flap that provided sufficient coverage to critical structures, adequate restoration of the region contour, and enough volume to offset potential adverse effects of subsequent radiation therapy. In addition, this report incorporates a concise literature review highlighting the expanding indications of the bipedicled DIEP flap beyond breast reconstruction, showing the versatility and efficacy of the bipedicled DIEP flap in addressing complex soft-tissue defects in various anatomical areas.

## Introduction

Since its initial report by Koshima and Soeda in 1989 [1], the deep inferior epigastric perforator (DIEP) flap has steadily gained popularity and is now widely regarded as the gold standard technique for autologous breast reconstruction. However, the utilization of the DIEP flap has not been limited to breast reconstruction alone. Numerous studies have extensively explored its application in reconstructive procedures involving the upper and lower extremities, head and neck, and axillary region, thus expanding the indications for this flap [2]. Although it may not be the typical first-line choice for defects in these areas, the DIEP flap’s ability to transfer a significant volume of tissue makes it a viable option for selected patients with large soft-tissue defects. Moreover, the flap’s coverage can be further extended by including its two vascular pedicles.

The bipedicled DIEP flap was initially introduced as a technique for breast reconstruction in patients with specific characteristics: a large/ptotic contralateral breast, a midline abdominal scar, and/or extensive radiation damage to the chest wall [3, 4]. In addition, this double-pedicled flap has been utilized in a few reported cases in non-breast areas, particularly for the reconstruction of lower limb defects [5–13]. Although conventional free flaps are typically suitable for addressing mid-to-large-sized defects in the cervico-thoracic region, certain unique cases involving larger defects may require a substantial, well-vascularized tissue that can provide sufficient coverage for critical structures. Furthermore, particularly in oncologic reconstruction, it is crucial to transfer enough volume to offset the potential detrimental effects of subsequent radiation therapy on the flap. In this context, we present a case involving a patient with a recurrent giant thyroid tumor who underwent surgical resection. The resulting extensive cervico-thoracic defect was reconstructed using a bipedicled DIEP flap.

## Case report

**Figure 1:**
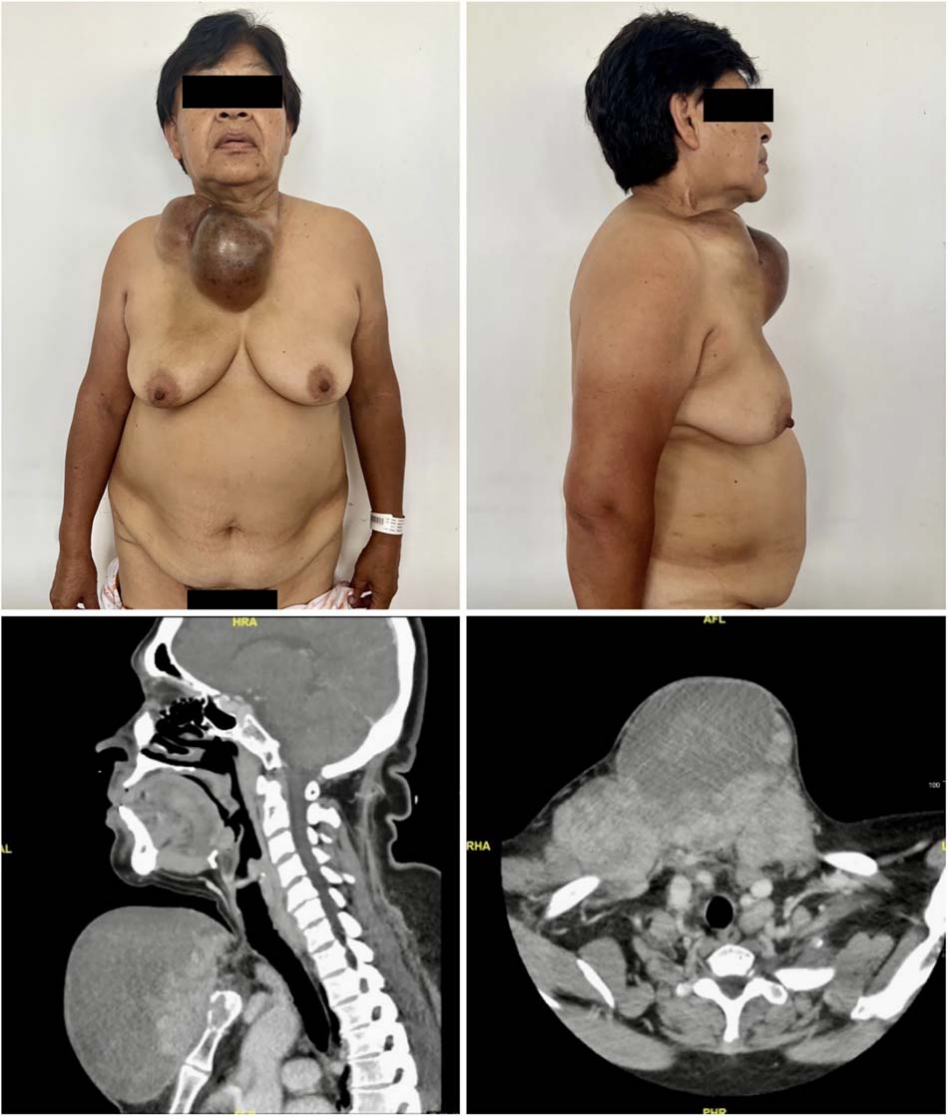
Preoperative photographs (upper, left and right) showing a large, hardened, bilobulated mass measuring 14 × 21 cm across the supraclavicular, infraclavicular, and presternal regions. The CT scans (lower, left and right) showing the posterior surface of the mass in contact with the pectoralis major muscle in the midline, the cortex of the sternal manubrium and the medial ends of both clavicles, and a poor interphase with the left sternocleidomastoid muscle at its caudal insertion.

A 69-year-old woman with history of total thyroidectomy and right neck dissection due to thyroid cancer 9 years before presented with a large, hardened, bilobulated mass measuring 14 × 21 cm. The tumor spanned across the supraclavicular, infraclavicular, and presternal regions and had been progressively growing over the course of 3 years, accompanied by pain. Fine needle aspiration biopsy of the tumor and cervical and axillary lymph nodes revealed papillary carcinoma. Computed tomography scans showed the posterior surface of the mass in contact with the pectoralis major muscle in the midline, as well as the cortex of the sternal manubrium and the medial ends of both clavicles. Likewise, there was a poor interphase with the left sternocleidomastoid muscle at its caudal insertion (Fig. 1). Multiple adenopathies were also detected in the cervical, thoracic, and axillary regions.

**Figure 2:**
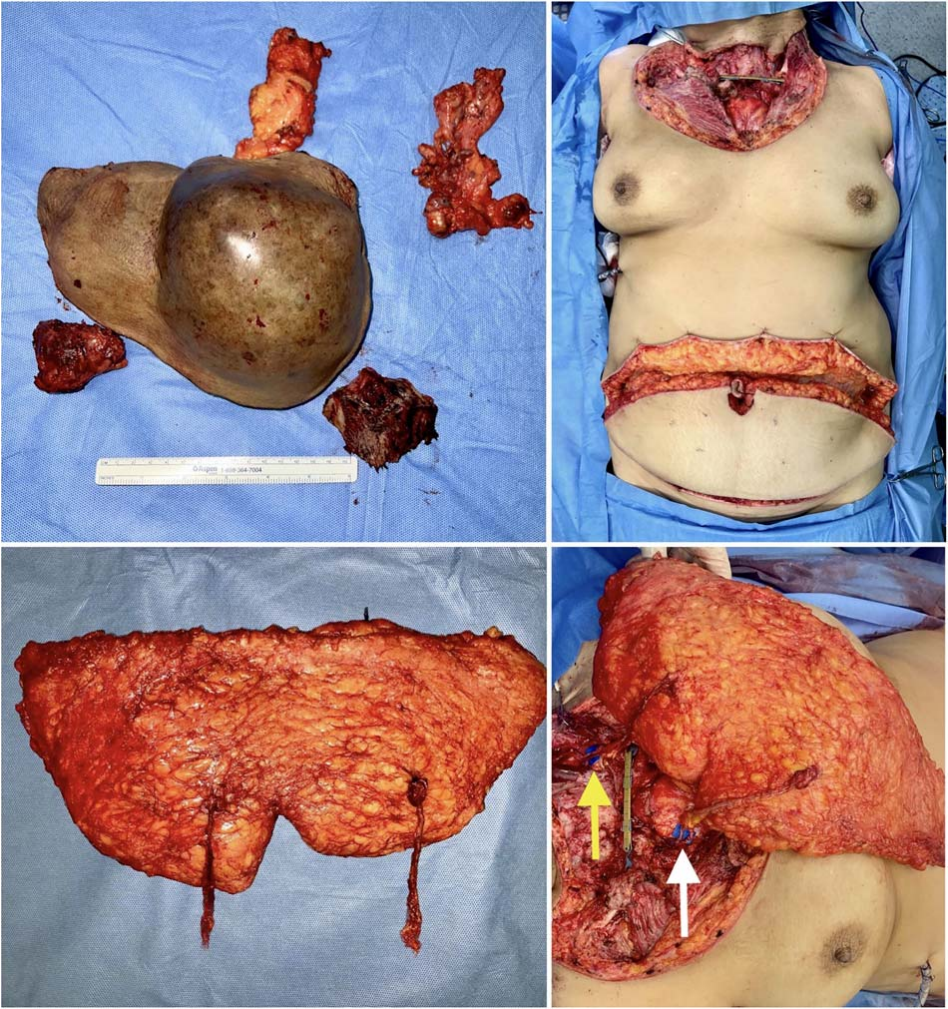
Intraoperative views of the specimens (upper, left), the surgical defect following resection measuring 16 × 27 cm (upper, right), the harvested bipedicled DIEP flap (lower, left), and after anastomoses to left transverse cervical vessels ( lower arrow) and right internal mammary vessels ( upper arrow) (lower, right).

**Figure 3:**
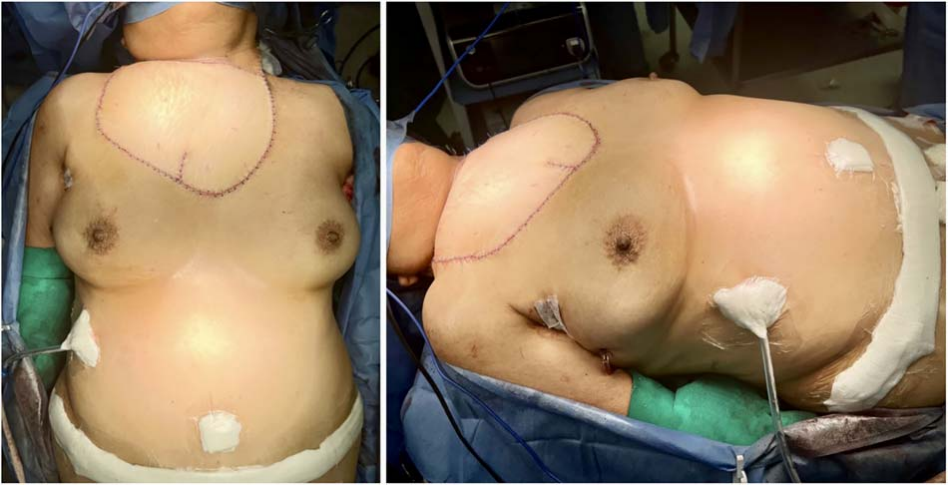
Immediate postoperative views depict sufficient coverage of the defect and satisfactory adaptation of the flap to the regional contour.

The ablative procedure involved resecting the tumor, performing a left neck dissection, bilateral axillary dissection, and partial excision of the manubrium and body of the sternum. The integrity of the right internal mammary vessels was successfully preserved. Thorax stabilization was performed with a pre-contoured locking compression titanium plate fixed at the clavicles. A chest tube was inserted and left in place for 3 days. The resulting soft tissue defect measured ~16 × 27 cm. Simultaneously with the resection, a bipedicled DIEP flap was harvested. After carefully dissecting the vessels in the recipient zone, the right internal mammary and left transverse cervical vessels were confirmed to be adequately preserved and were anastomosed to the left and right flap pedicles, respectively (Fig. 2). Once satisfactory vascular patency was ensured, the flap was shaped to match the dimensions of the defect and provide adequate coverage of critical cervico-thoracic structures, restoring the contour of the region (Fig. 3). No vascular-related complications were developed within the early postoperative period. The patient began sitting on the third day after surgery and gradually resumed walking on the fourth day. The flap survived completely, and the surgical incisions healed uneventfully total hospitalization length was 16 days.

**Figure 4:**
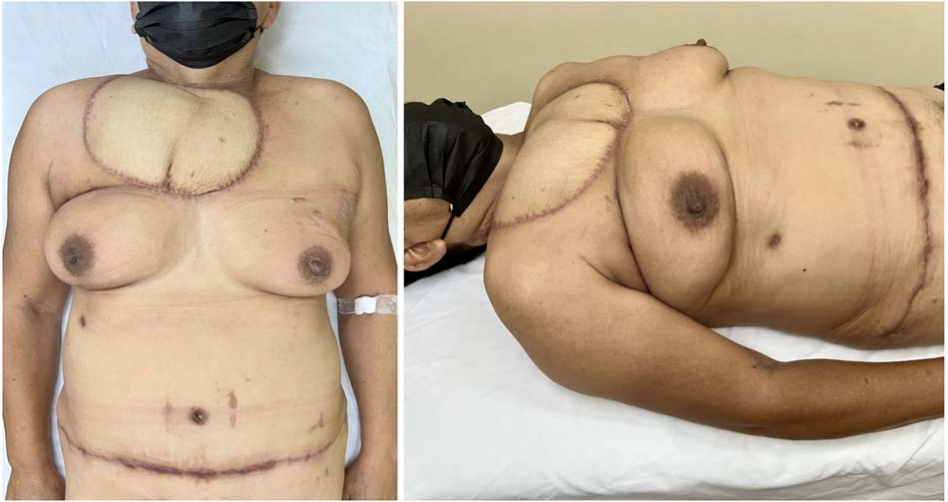
Appearance at 3-months postoperatively showcases a well-perfused flap with favorable scarring, and the absence of bulging or weakness in the abdominal wall at the donor site.

On follow-up3 months after surgery, the flap showed healthy, well-perfused tissue with good scarring, resulting in a good esthetic result. The donor site demonstrated no signs of bulging or abdominal wall weakness, and the patient reported no discomfort or pain (Fig. 4).

## Discussion

The bipedicled DIEP flap initially emerged as a technique exclusively intended for breast reconstruction, targeting specific patient populations with unique characteristics. The inclusion of both inferior epigastric pedicles facilitates the transfer of the entire abdominal skin and fat, allowing for effective reconstruction in cases involving a large contralateral breast or the presence of a midline abdominal scar. Nevertheless, the application of this flap has extended to non-breast areas, offering viable solutions for challenging extensive soft-tissue defects in other anatomical regions. The bipedicled DIEP flap, owing to its expansive surface area and minimal donor-site morbidity, has been successfully used in the reconstruction of substantial defects in the upper and lower extremities, head and neck, and axillary region. Although muscle flaps were conventionally used to address defects in these areas, the advent of perforator flaps has allowed the transfer of comparable or even larger tissue volumes without the associated morbidity at the donor site. Table 1 presents a comprehensive compilation of pivotal articles that delve into the utilization of the bipedicled DIEP flap in non-breast regions.

**Table 1:** Articles reporting the use of bipedicled DIEP flap for non-breast reconstruction

References	Defect localization	Number of cases	Intraflap anastomoses	Vascular-related complications	Follow up	Final outcome
Mahajan *et al.* (2016)	Upper and lower limb	12	7/12	Partial flap necrosis 3/12Complete flap necrosis 1/12	Not reported	91.6% successful
Van Landuyt *et al.* (2006)	Lower limb	9	4/9	Partial flap necrosis 1/8 Complete flap necrosis 1/9	Not reported	87.5% successful
Qing *et al.* (2019)	Lower leg	1	No	None	36 months	Successful
Grinsell *et al.* (2012)	Thigh	1	Yes	None	2 weeks	Successful
Melnikov *et al.* (2020)	Lower leg	1	No	None	6 months	Successful
Bota *et al.* (2017)	Thigh	1	No	None	24 weeks	Successful
Slater *et al.* (2014)	Scalp	1	No	None	7 months	Successful
D’Arpa *et al.* (2019)	Axilla	1	Yes	None	64 months	Successful
Jin *et al.* (2009)	Facial lower third	1	No	None	4 months	Successful

Abbreviations: DIEP, deep inferior epigastric perforator

Mahajan *et al.* [5] studied 12 male patients with large extremity defects, using bipedicled DIEP flaps. Among the flap procedures, five involved bipedicled DIEP flaps with two sets of recipient vessels, whereas others used intraflap anastomosis. Complications included partial necrosis and dehiscence in two flaps, requiring venous revision in two cases, and one complete flap necrosis. The patients did not experience bulging, hernia, seroma, or infection. Similarly, Van Landuyt *et al.* [6] reported on nine patients who underwent lower extremity reconstruction with bipedicled DIEP flaps, with complications limited to partial and complete flap necrosis in one case each. Case reports have also shown the efficacy of this flap in extensive lower extremity reconstruction in both adult and pediatric patients [7–10].

Likewise, other studies have documented the use of the bipedicled DIEP flap for addressing defects in the head and neck region. Slater *et al.* [11] presented a case report involving a patient with a severe cranial deformity compounded by an indolent infection resulting from infected hardware used in a prior repair of traumatic injuries. The reconstruction approach employed a staged, two-step procedure utilizing a bipedicled DIEP flap for coverage. This approach allowed for the complete eradication and treatment of the infection before the delayed insertion of a cranioplasty for the reconstruction of the cranial defect. For their part, Jin *et al.* [12] reported a simultaneous procedure involving facial scar repair and oral aperture opening using a windowed, bilateral, bipedicled DIEP flap in a 20-year-old male patient who had experienced severe postburn scars on the face and neck, accompanied by significant cicatricial microstomia. The results of the procedure demonstrated a remarkable esthetic and functional outcome. Finally, D’Arpa *et al.* [13] reported on 12 cases treated for axillary hidradenitis suppurativa using DIEP flaps, with one of the cases involving the utilization of a bipedicled DIEP flap due to the size of the defect.

The extensive size, specific location, and exposure of critical anatomical structures posed unique challenges in addressing the defect presented by our patient. Utilizing pedicled options would have required the use of at least two local/regional flaps, potentially leading to donor site morbidity and an unsatisfactory esthetic outcome. A free latissimus dorsi flap typically proves effective in addressing the majority of defects on the thoracic wall. However, extensive defects may require its transfer as a muscle-only flap plus skin grafting, potentially delaying radiation therapy and ultimately yielding suboptimal esthetic outcomes. Moreover, especially in older patients, harvest of unilateral latissimus dorsi flap has been associated with reduction in stability of spinal posture in the long term [14]. Although a double anterolateral thigh or profunda artery perforator free flap could have been considered given the presence of multiple recipient vessels in the affected area, our assessment determined that a single flap with adequate tissue would provide optimal coverage for the exposed vascular and bony structures. This approach resulted in a more natural contour restoration while minimizing the morbidity associated with utilizing two different donor sites. Moreover, the redundancy of skin and adipose tissue in our patient’s infraumbilical area facilitated a tension-free closure of the abdomen.

This case report underscores the effectiveness of the bipedicled DIEP flap in addressing a large cervico-thoracic defect, constituting, to the best of our knowledge, the first documentation of using a bipedicled DIEP flap for reconstruction in this anatomical region. Consequently, this approach introduces a new strategy that enriches the reconstructive surgeon armamentarium. As the field of reconstructive microsurgery continues its relentless evolution, this flap shows promising potential in addressing extensive soft-tissue defects across diverse anatomical regions, offering adequate functional and esthetic results while minimizing donor-site morbidity.

## Data Availability

The data that support the findings of this study are available from the corresponding author upon reasonable request.
